# Quantifying urban growth patterns in Hanoi using landscape expansion modes and time series spatial metrics

**DOI:** 10.1371/journal.pone.0196940

**Published:** 2018-05-07

**Authors:** Duong H. Nong, Christopher A. Lepczyk, Tomoaki Miura, Jefferson M. Fox

**Affiliations:** 1 Faculty of Environment, Vietnam National University of Agriculture, Hanoi, Vietnam; 2 East-West Center, Honolulu, HI, United States of America; 3 School of Forestry and Wildlife Sciences, Auburn University, Auburn, AL, United States of America; 4 Department of Natural Resources and Environmental Management, University of Hawaii at Manoa, Honolulu, HI, United States of America; Rice University, UNITED STATES

## Abstract

Urbanization has been driven by various social, economic, and political factors around the world for centuries. Because urbanization continues unabated in many places, it is crucial to understand patterns of urbanization and their potential ecological and environmental impacts. Given this need, the objectives of our study were to quantify urban growth rates, growth modes, and resultant changes in the landscape pattern of urbanization in Hanoi, Vietnam from 1993 to 2010 and to evaluate the extent to which the process of urban growth in Hanoi conformed to the diffusion-coalescence theory. We analyzed the spatiotemporal patterns and dynamics of the built-up land in Hanoi using landscape expansion modes, spatial metrics, and a gradient approach. Urbanization was most pronounced in the periods of 2001–2006 and 2006–2010 at a distance of 10 to 35 km around the urban center. Over the 17 year period urban expansion in Hanoi was dominated by infilling and edge expansion growth modes. Our findings support the diffusion-coalescence theory of urbanization. The shift of the urban growth areas over time and the dynamic nature of the spatial metrics revealed important information about our understanding of the urban growth process and cycle. Furthermore, our findings can be used to evaluate urban planning policies and aid in urbanization issues in rapidly urbanizing countries.

## Introduction

Today, more than half of the world’s population live in urban areas [1, 2] and is expected to continue growing in the future. In order to accommodate this urban growth land is transformed from one land use or land cover to a form of urban land. This transformation (i.e. urbanization) is one of the most powerful, irreversible, and visible impacts humans have on the environment [3, 4]. Arguably, urbanization provides many beneficial aspects to human society, such as boosting the economy, enhancing society, providing cultural and educational centers, and increasing economic productivity. However, urbanization also leads to significant landscape and ecosystem transformations and creates a host of problems, including environmental pollution, waste disposal, energy inefficiency, inadequate housing and water supplies, increase risk of communicable diseases, and weakening of social support systems. Considering the large impacts of urbanization on the world’s ecosystems and human population it is critical to understand patterns of urbanization in order to aid in urban planning and improve natural resource management.

The spatial arrangement (i.e. patterns) of urban areas in a particular time period often provides a reflection of various social, economic, and political factors that influenced land-use decisions [5]. Understanding these patterns and processes thus requires understanding how cities are spatially growing and organized, how urban growth results in a specific pattern, and ultimately what will be the consequences of such patterns and processes [6]. Given this need a number of theories have been put forth to describe the dynamics of urban patterns, including the Concentric Zone Theory [7], Sector Theory [8], Multiple Nuclei Theory [9], and the Wave Theory Analog Approach [10]. While these theories have provided a better understanding of urban structure and dynamics and have been widely used to model urban systems they have not been adequately tested to address spatiotemporal dynamics of urban patterns [11, 12]. However, advances in computational power, increased availability of remotely sensed data and landscape pattern analysis tools provide a powerful means for modeling and testing these urban morphological theories [13, 14]. Landscape patterns analysis typically involve spatial metrics to quantify spatial characteristics at both patch and landscape levels. As such, these spatial metrics can help to inform the process of urban development at various scales and be used to develop, improve, and test urban growth models [15, 16].

Many urban growth models conceptualize growth dynamics as occurring in phases or cycles, rather than linearly in space or time [12]. As a result it is difficult to model the complexity of the urban region in a single model. However, one model that provides a useful conceptual model of spatiotemporal urban growth dynamics is the diffusion-coalescence cycle [11, 15]. The term ‘diffusion’ refers to the dispersed expansion of new urban areas from the origin point or ‘seed’ location [11] and is represented by the dominance of ‘edge expansion’ and ‘spontaneous growth’ [17, 18]. Meanwhile, the term ‘coalescence’ refers to the union of individual urban patches [11] or the ‘infilling’ of open spaces within the urban complex [17, 18]. Three urban growth processes (infilling or gap-filling, edge expansion, and spontaneous or outlying growth) have been widely discussed in the literature [17, 18] and they often occur concurrently, but the abundance of each type varies within the urbanization cycle and thus determines the urban growth phases [19, 20].

Because infilling, edge expansion, and spontaneous growth have been demonstrated to provide a valuable approach to address urban growth phases, our overarching goal was to use these characteristics in conjunction with time series of spatial metrics to quantify urban growth patterns in a rapidly urbanizing area of the world. To address this goal we had the following objectives: 1) quantify the growth rates, growth modes, and resultant changes in landscape patterns of urbanization by using spatial metrics [21] and landscape expansion modes [17, 18]; and, 2) evaluate the extent to which the process of urban growth conforms to the diffusion-coalescence hypothesis [11, 15]. We used Hanoi, Vietnam as a model urban area given its rapid rate of urbanization.

## Materials and methods

### Background of the study area

**Fig 1:**
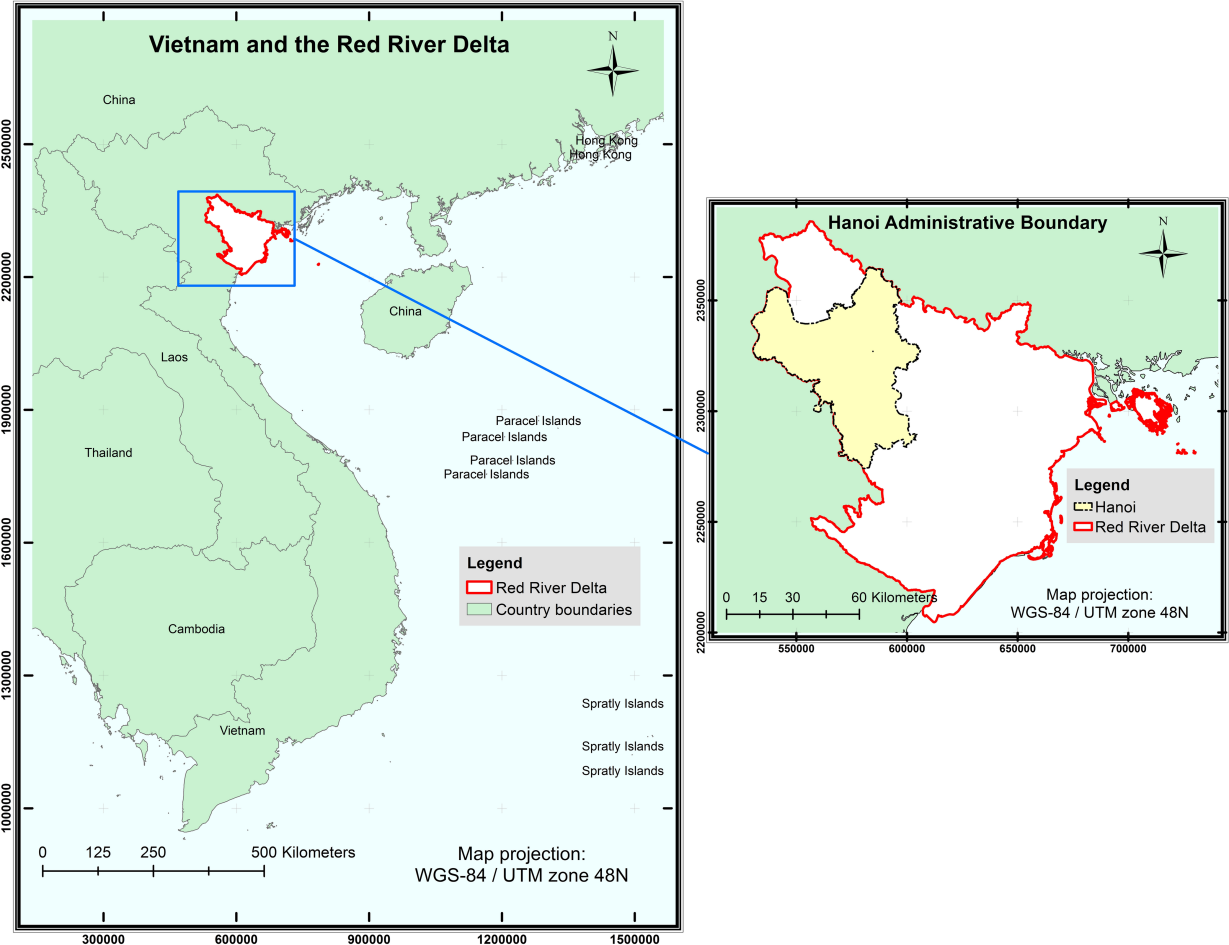
Maps of study area. Shapefile reprinted from GADM database under a CC BY license, with permission from Global Administrative Areas (www.gadm.org). The figure was made with ArcGIS 10.3 under a CC BY license, with permission from ESRI (www.esri.com).

Hanoi is the capital city of Vietnam (Fig 1) and has experienced considerable transformation in both socio-economic and physical forms since the ‘Doi Moi’ reform policy in 1986 [22]. The annual GDP growth rate of Hanoi was 10.7%, substantially higher than the national rate of 7.1% during 1995–2000 [23], and increased three-fold between 2000 and 2008 [24]. Along with the economic growth, the city’s population has been growing at 3% annually, reaching 3.2 million in 2007 [25]. As a result, the city expanded its administrative boundary in 2008, doubling the population of the city-region to 6.4 million, thereby making it the second largest city in Vietnam after Ho Chi Minh City [24].

The inflow of Foreign Direct Investment has promoted rapid urbanization and industrialization, concentrated primarily around the city, with large tracts of agricultural land being reserved for industrial and urban development projects [26]. Among nine economic regions in Vietnam, the Red River Delta is the only region that showed a net loss of agricultural employment between 1999 and 2009 [26]. According to Hanoi’s 2000–2010 land-use plan, about 11,000 ha of land, mostly annual crop land, were converted for 1,736 projects, causing the loss of 150,000 farming jobs [27].

The rapid conversion of agricultural land to urban uses has raised a concern about food provision for urban populations and livelihoods of peri-urban famers [28]. The encroachment of urban areas into agricultural areas also resulted in fragmentation and isolation of farmlands [28, 29], resulting in negative impacts on crop productivity, increasing labor and other expenses, and preventing the use of modern and mechanized equipment [30, 31]. Because agricultural land is finite and there is also competing interests in what to use the land for (e.g., industrial and infrastructural projects), agricultural land should be broadly classified on the basis of ecological conservation and economic efficiency [32]. The rapid urbanization and loss of agricultural land in the Red River Delta are challenging the government’s ability to manage the rural-urban transition effectively. There is thus an urgent need to explore the urban growth patterns and their spatial characteristics.

### Data sources

**Fig 2:**
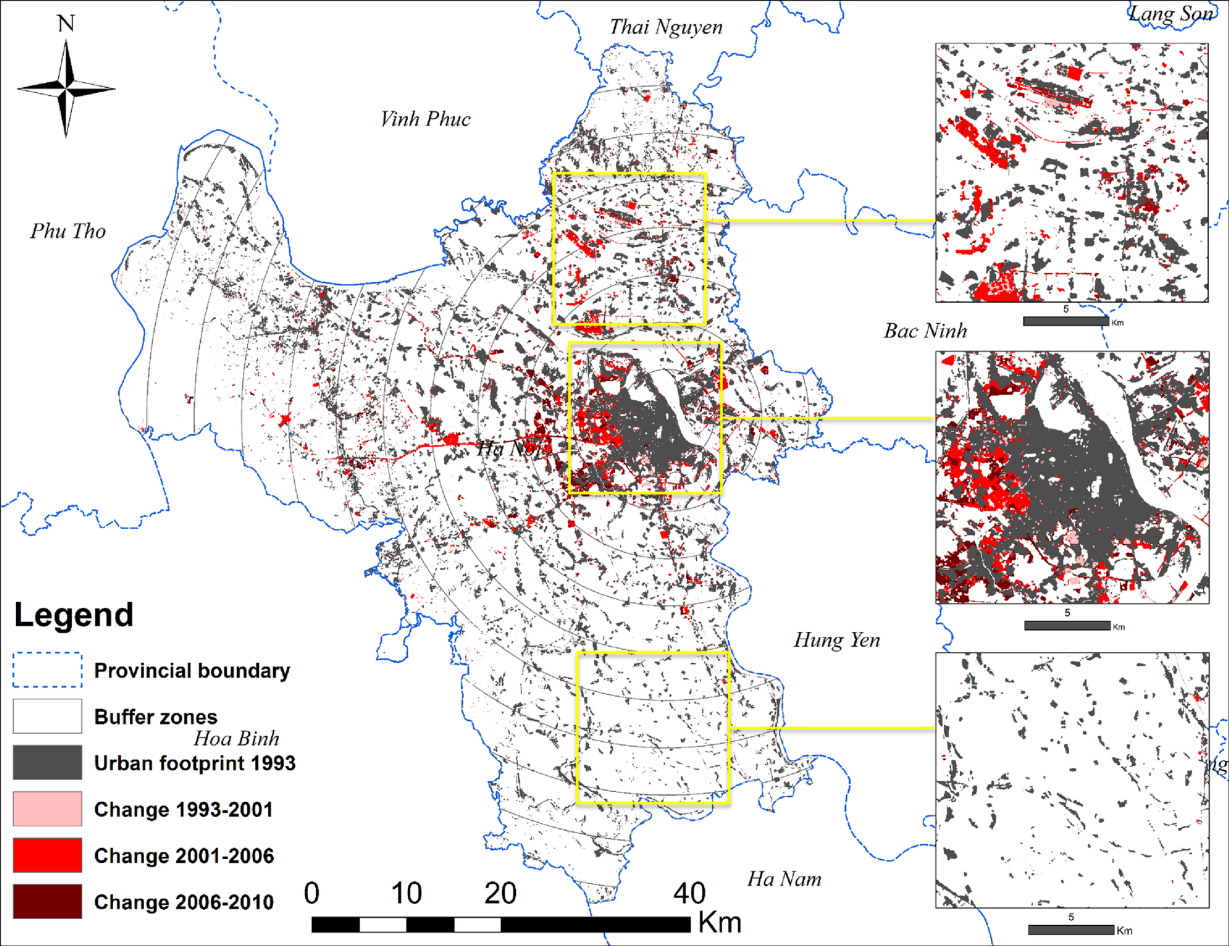
Hanoi mapped by changes in built-up areas over time across the 5 km buffers from the city center. Shapefile reprinted from GADM database under a CC BY license, with permission from Global Administrative Areas (www.gadm.org). The figure was made with ArcGIS 10.3 under a CC BY license, with permission from ESRI (www.esri.com).

Nong et al. [33] used multi-temporal image stacks of Landsat images to classify land cover in Hanoi into seven classes: agriculture, urban footprint, forest, water, and three change classes (agriculture to built-up) between the time periods of 1993–2001, 2001–2006, and 2006–2010. There is concern that seasonal variations in spectral reflectance could potentially result in confusion between built-up areas and fallow farmland at certain time periods in the remote sensing classification because both built-up areas and fallow farmland show high reflectance in the visible-infrared wavelength regions. However, Nong et al. [33] have successfully distinguished changes within agricultural land use types and changes from agricultural land to built-up land using multi-temporal image stacks of Landsat images for land cover classification of Hanoi City. The approach considers that if a field is converted to built-up land, this change should be “confirmed” in subsequent satellite images because the transformation from agriculture to built-up land is generally unidirectional. We used the existing land cover data developed in Nong et al. [33] to study the spatial characteristics of urban land expansion in Hanoi. Specifically, we focused on the spatial characteristics of the urban footprint in 1993, and urban patches that were converted from agriculture to built-up land in three periods: (i) 1993–2001, (ii) 2001–2006, and (iii) 2006–2010 (Fig 2).

### Rural-urban gradient analysis

From its early development, Hanoi has followed a monocentric city in urban structures introduced by French colonist and later influenced by the central planning model after independence [34]. Hanoi’s Old Quarter is the oldest continuously developed area of the city [35] and from there it has expanded over time. Therefore, to quantify the scale and impact of urbanization, we adopted a rural-urban gradient analysis by using a multiple buffer zone system. First, we defined a center point located in Hoan Kiem district in the Old Quarter because this is one of the first urban districts of Hanoi and is often referred to as the center of the city [36, 37]. From the center point, we then created multiple buffer zones at 5 km intervals until we covered the entire Hanoi boundary (Fig 2).

We defined the 5 km interval buffer zones because the four initial urban districts in 1993 (the beginning of the study period) were encompassed within a radius of 5 km. Within each buffer zone, we calculated annual urban growth rates and analyzed six patch metrics (patch density, edge density, landscape shape index, largest patch index, area-weighted mean Euclidean nearest-neighbor distance, and area-weighted mean patch fractal dimension; Table 1) and three landscape expansion modes (infilling, edge-expansion, and spontaneous growth) for the three time periods to understand how they might change across the gradient from city center outward. Because of the difference in area among the buffer zones, we standardized all measures by their mean values and percentages to make them comparable. To deal with patches that lie between two buffer zones, we did not include the buffer zones in our spatial metric calculations in FRAGSTATS. The buffer zones were used only after metrics of each patch were calculated. After that we created centroid points for each patch, so if a patch lies between two buffer zones, its metrics will belong to the buffer zone where the centroid is located.

**Table 1:** Patch metrics selected for spatiotemporal analysis.

No	Patch metrics	Range	Description	Diffusion	Coalescence
1	Patch Density (PD)	PD ≥ 0, no limit	Number of patches per 100 hectare.	Increasing	Decreasing
2	Edge Density (ED)	ED ≥ 0, no limit	Sum of the lengths (m) of all edge segments in the landscape per hectare.	Increasing	Decreasing
3	Landscape Shape Index (LSI)	LSI ≥ 1, no limit	Normalized ratio of edge to area that measures the shape complexity of a specific class or the whole landscape.	Increasing	Decreasing
4	Largest Patch Index (LPI)	0 < LPI≤100	Proportion (%) of the landscape comprised by the largest patch.	Decreasing	Increasing
5	Area-weighted Mean Euclidean Nearest-Neighbor Distance (ENN_AM)	ENN_AM ≥ 0, no limit	Distance from a patch to a neighboring patch of the same or different class, based on the nearest cell center-to-cell center.	Increasing	Decreasing
6	Area-weighted Mean Patch Fractal Dimension (FRACT_AM)	1≤ FRACT_AM ≤2	Complexity of a patch by a perimeter area proportion.	Increasing	Decreasing

To quantify how landscape patterns changed over time we computed the difference in each patch metric across the three time periods as the difference in *patch metric i = patch metric i (t*_*2*_*)–patch metric i (t*_*1*_*)*. For a given patch metric, an increase in its value from *t*_*1*_ to *t*_*2*_ leads to a positive difference, whereas a decrease in its value from *t*_*1*_ to *t*_*2*_ results in a negative difference.

### Hypothetical model of urban growth

**Fig 3:**
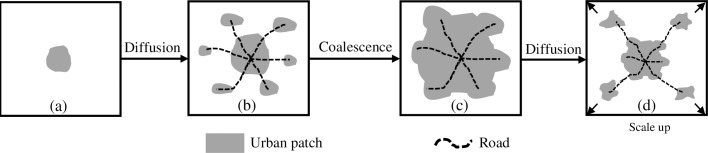
Hypothetical sequence of the spatial evolution of an urban area.

Patterns and processes in the agricultural to urban landscape transitions in Hanoi were characterized by comparing them to a hypothetical model of spatial evolution [15]. In the spatial evolution model, urban land area increases through a combination of diffusion and coalescence processes (Fig 3). In the hypothetical model, a compact urban landscape (Fig 3A) becomes more fragmented over time as new developments are established in the periphery of an urban core (Fig 3B). As they expand in size, these patches begin to aggregate until most of the land has become urbanized (Fig 3C) and the next cycle of diffusion–coalescence begins as the urban scales up (Fig 3D). We quantified and tracked these changes by examining spatiotemporal patterns of urban patches through their spatial metrics and urban growth typologies.

### Measuring urban growth

We calculated the annual growth rate of urbanized land as follows:

$$r=\frac{1}{t_{2}-t_{1}}\ln\frac{A_{t2}}{A_{t1}}$$ (1)

where *A*_t2_ and *A*_t1_ are the built-up land area in year *t*_2_ and year *t*_1_, respectively. Eq 1 has been widely used to calculate the annual rate of forest change [38] as well as the annual rate of urban growth [5]. Furthermore, Eq 1 assumes urban growth is an exponential process that is mathematically identical to the annual rate of compound interest.

### Typologies of urban growth

**Fig 4:**
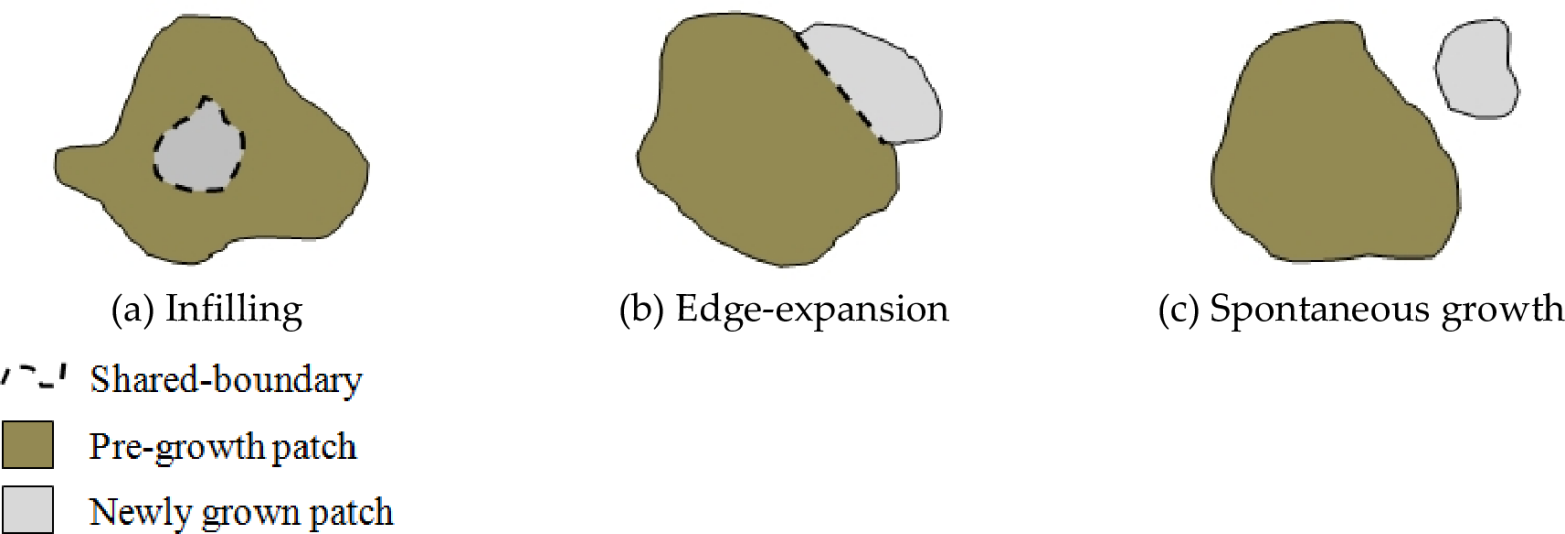
Three urban growth types. (a) Infilling growth which occurs within urbanized open space and increases contiguity of built-up area by filling in that space; (b) Edge expansion which refers to non-infill development intersecting the urban footprint and extending outward from previous development; and, (c) Spontaneous growth which neither intersects the urban footprint nor is contiguous with previously developed areas and has greatest effect on fragmentation of open lands.

In addition to the relationship between newly grown urban patches and pre-growth urban patches, Xu et al. [18] proposed a quantitative method to distinguish three urban growth types: infilling, edge expansion, and spontaneous growth. The dominance of each growth types is meaningful to describe the process of landscape pattern changes between two or more time points [19, 20]. Whether a growth patch is called infilling, edge expansion, or spontaneous growth is determined by LEI value which is calculated as follows:

$$LEI=\frac{L_{C}}{P}$$ (2)

where *LEI* is Landscape Expansion Index, *L*_*C*_ is the length of the common boundary of a newly grown urban patch and the pre-growth urban patches, and *P* is the perimeter of this newly grown patch. Urban growth type is identified as (a) infilling when LEI > 0.5, (b) edge-expansion when 0 < LEI ≤ 0.5, and (c) spontaneous growth when LEI = 0, which indicates no shared-boundary (Fig 4).

To determine the relative dominance among the different forms of urban growth across a landscape or over time, Liu et al. [17] developed an Area-Weighted Mean Expansion Index (AWMEI), calculated as follows:

$$AWMEI=\sum_{i=1}^{N}LEI_{i}*\left(\frac{a_{i}}{A}\right)$$ (3)

where *LEIi* is the LEI value for a newly growth patch *i*, *α*_*i*_ is the area of this new patch, and *A* is the total area of all these newly grown patches. Larger values of AWMEI correspond to more compact form of urban growth while smaller values of AWMEI imply the prevalence of leapfrogging or spontaneous development or urban sprawl. An increase of AWMEI over time signifies a coalescence phase while a decrease of AWMEI signifies a diffusion phase.

### Quantifying spatiotemporal patterns of urbanization using spatial metrics

The literature is replete with a very large number of landscape metrics that can be considered for spatial analysis. However, many landscape metrics are highly correlated with one another [39, 40] and therefore redundant. In fact, Riitters et al. 1995 [41] analyzed 55 different metrics and found only five independent factors. Thus, not all spatial metrics are measuring different qualities of spatial patterns. In addition, the temporal scale is an important factor to consider when selecting the appropriate spatial metrics because the harmonic oscillation behaviour of metrics are different. In the case of evaluating urban growth patterns a variety of specific metrics have previously been used. For instance, Dietzel et al. 2005 [11] used 12 metrics for a case study in Houstan Metropolitan Area and found that only seven presented harmonic oscillation during the 28 year study period. However, as Alberti et al. 2000 [42] note, there is no set of unique metrics to urban environments. Considering these points we used a set of spatial metrics that were best suited for cities like Hanoi. Specifically, we based our selection on a set of spatial metrics which possess explicit meanings in relation to the diffusion and coalescence processes of urban growth [11, 19, 43, 44]. We then examined these metrics to make sure that they were sensitive enough to demonstrate harmonic behaviour over a 17 year time period of the land cover data. Based on these considerations we selected seven metrics to characterize the spatiotemporal patterns of Hanoi City. These seven selected metrics were patch density (PD), edge density (ED), landscape shape index (LSI), largest patch index (LPI), area-weighted mean Euclidean nearest-neighbor distance (ENN_AM) and area-weighted mean patch fractal dimension (FRACT_AM) (Table 1). Notably, how the patch metrics change over time can support either diffusion or coalescence models. All patch metrics were calculated in FRAGSTATS version 4.3 [45].

## Results

### Annual urban growth

**Fig 5:**
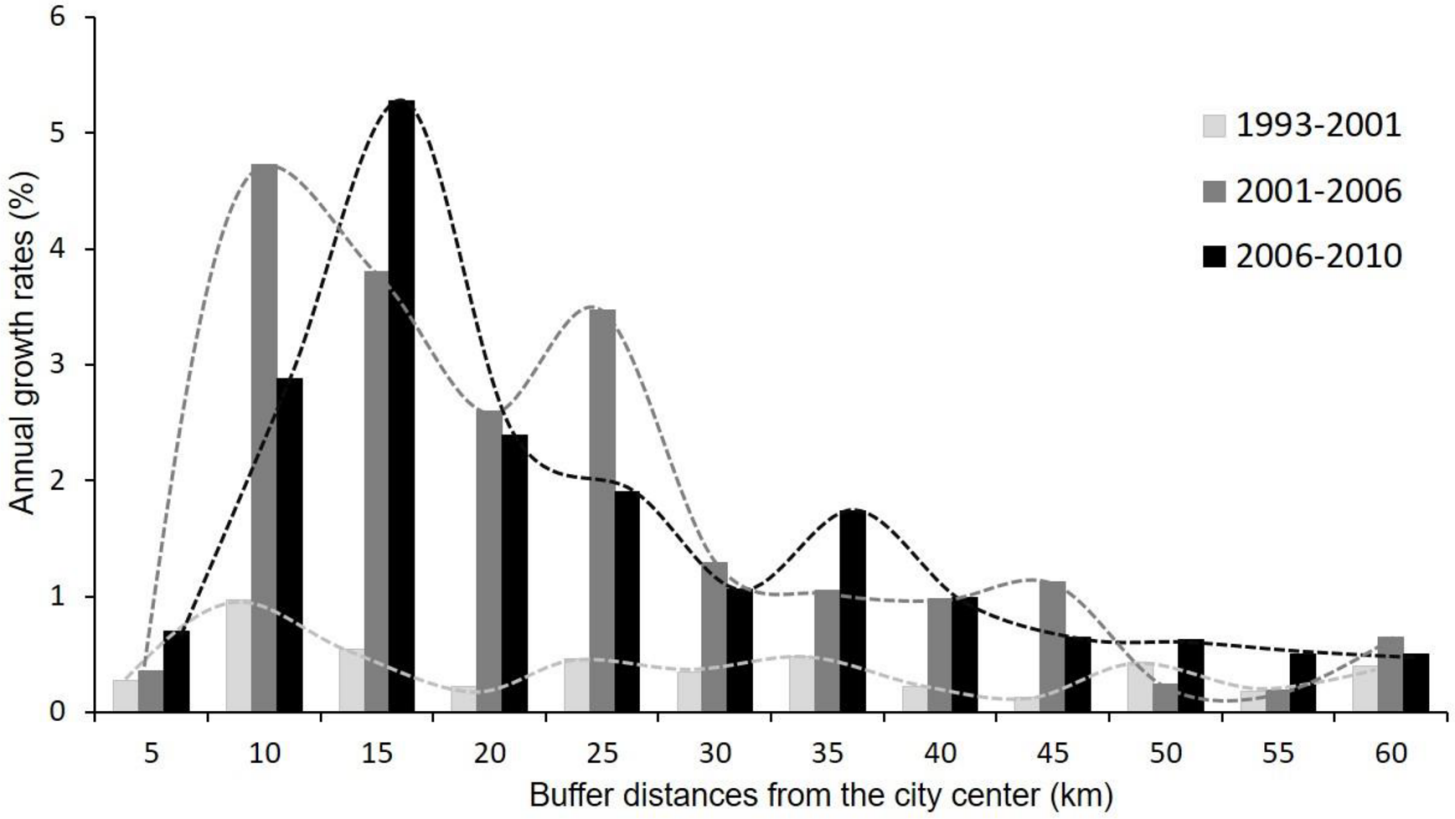
Annual growth rates of built-up area across different buffer zones.

From 1993 to 2010, the built-up land of Hanoi increased from 504 km^2^ (15% of the total land area) to 631 km^2^ (20% of the total land area). The annual growth rates of built-up land for the three time periods and for the 12 buffer zones showed that the rate of urbanization differed among the three periods and depended on the distance away from the urban center. The annual growth rates ranged from 0.12% to 5.27%, with higher growth rates found in the last two time periods (2001–2006 and 2006–2010) and at a distance of 10 to 35 km from the city center (Fig 5).

### Urban growth modes

**Fig 6:**
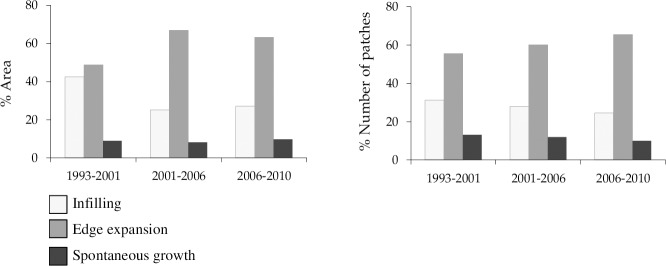
Percentages of infilling, edge expansion, and spontaneous growth areas and their percentage number of patches in the three time periods for Hanoi City.

Over the 17 year period the relative dominance of infilling, edge expansion, and spontaneous growth changed whereas both new urban patches emerged and the old ones expanded. These changes led to urban clusters enlarging and coalescing, ultimately forming the Hanoi urban agglomeration. Across all time periods the landscape was dominated by edge expansion, followed by infilling growth in terms of both the area and number of patches (Fig 6). Infilling growth decreased and edge expansion growth increased, whereas spontaneous growth remained relatively the same.

**Fig 7:**
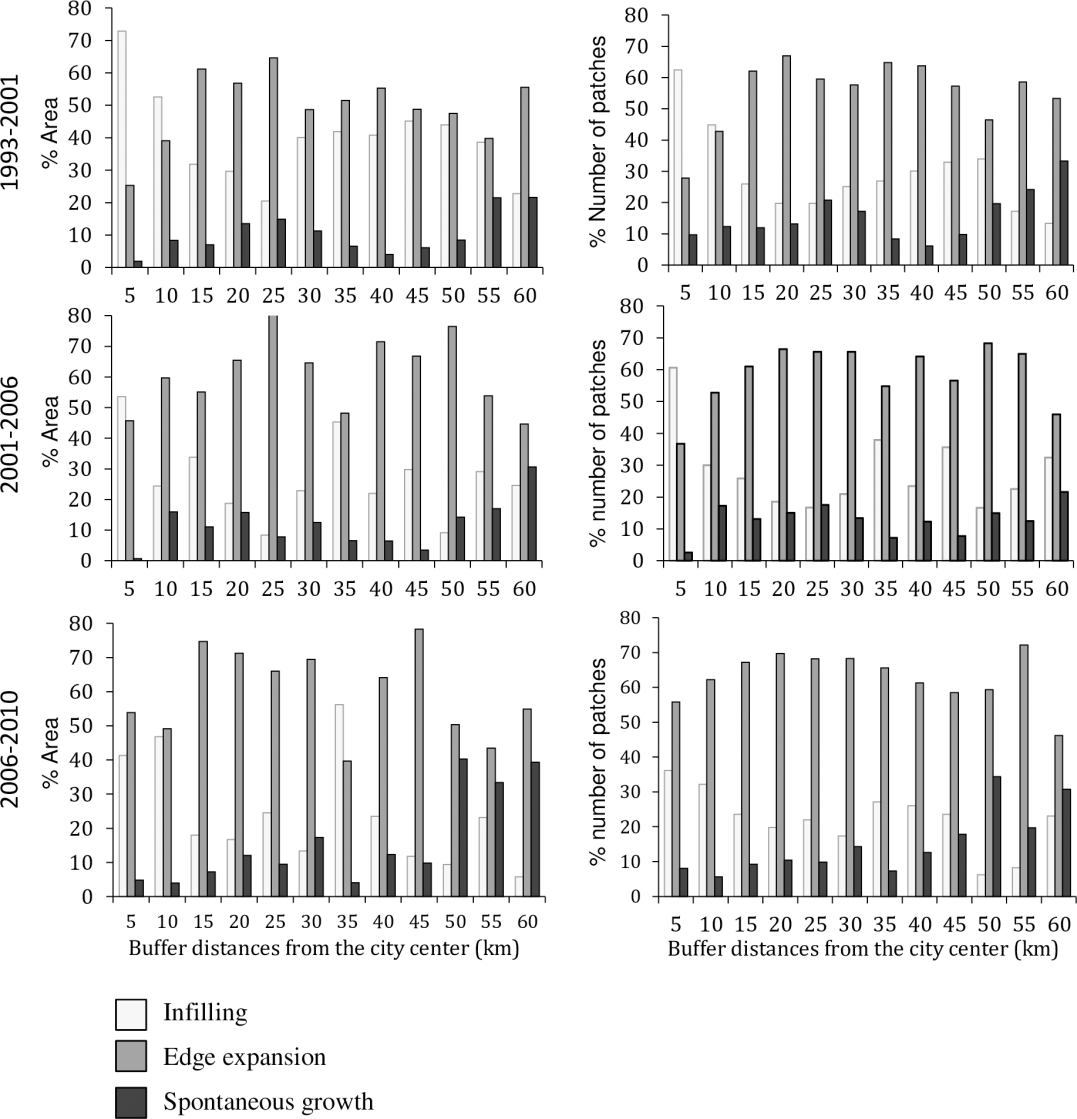
Percentages of infilling, edge expansion and spontaneous growth areas and their percentage number of patches in three time periods for twelve buffer zones.

Across the urban landscape, the relative dominance of growth patterns was different across space and time (Fig 7). For example, in the first period (1993–2001), the 5 and 10 km buffer zones were dominated by the infilling growth mode while the other buffer zones were dominated by the edge expansion growth mode. In the second period (2001–2006), the major urban growth mode of the 10 km buffer zone had shifted from the infilling to the edge expansion, while the infilling still dominated in the 5 km buffer zone. However, in the third period (2006–2010) the dominance of urban growth mode in the 5 km buffer zone shifted to the edge-expansion and the 35 km buffer zone exhibited a reverse trend where it shifted from the edge-expansion to infilling growth mode. Thus, there were temporal switches in urban growth modes in the 5, 10 and 35 km buffer zones over the three periods.

At different time periods, the relative dominance of the three growth modes in each buffer zone also showed different proportions between the patch area and patch number (Fig 7). For example, in the period 2006–2010, at the 35 km buffer zone, the infilling grew in a larger area but had a smaller number of patches than the edge-expansion growth mode. This landscape with a small patch area and a large patch number indicates a fragmented landscape. Therefore, the temporal shifts in relative dominance of three urban growth modes revealed by patch number may differ from those revealed by patch area (Fig 7). These two measures complemented each other, therefore providing more information when examined together.

**Fig 8:**
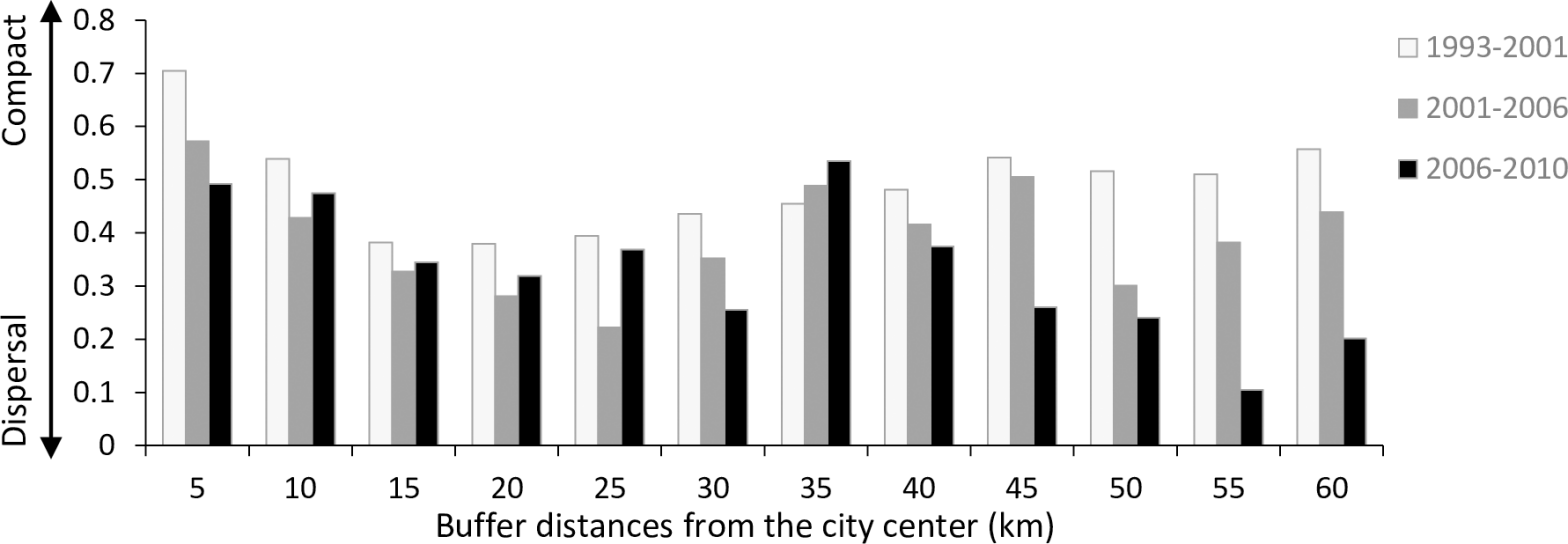
Spatial and temporal profiles of the area-weighted mean expansion index for twelve buffer zones.

The spatial and temporal profiles of the AWMEI (Fig 8), averaged for all communes in each buffer zone, showed three particular trends. First, the AWMEI decreased over time in the 5, 30, and 40 to 60 km buffer zones. Second, at the 35 km buffer zone, in contrast, the AWMEI increased over time. Finally, in the 10 to 25 km buffer zones, the urban morphologies revealed a distinct trend where all their AWMEI values decreased in the second period (2001–2006) but then increased again in the third period (2006–2010). Change of AWMEI over time indicated that the city has gone through the diffusion phase in the 5, 30, and 40 to 60 km buffer zones and the coalescence phase in 35 km buffer zone. From 10 to 25 km buffer zones, due to the high rate of urbanization the city has gone through both diffusion and coalescence phases.

### Landscape structures and changes during urbanization

**Fig 9:**
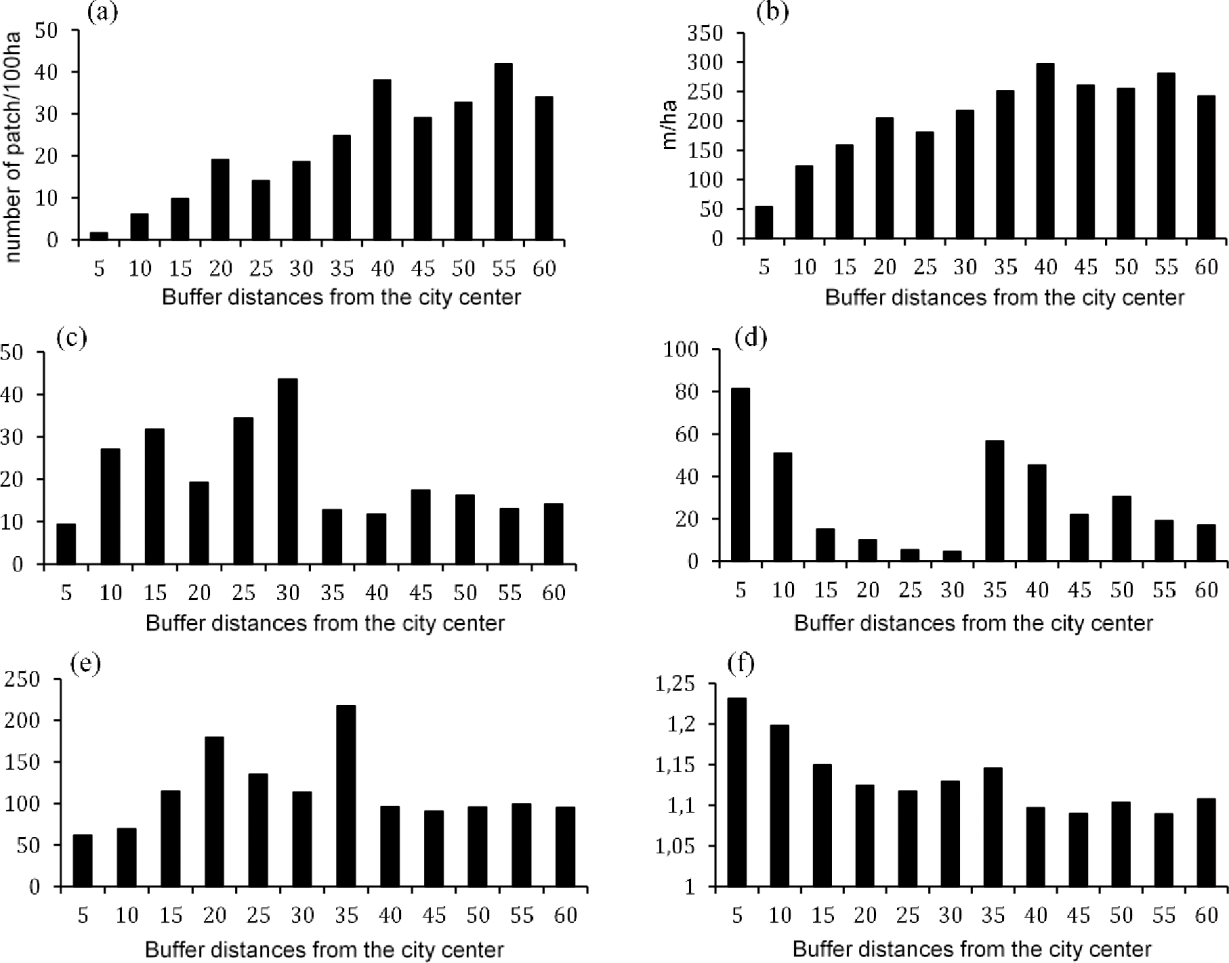
Mean selected patch metrics showing general landscape patterns over three time periods and across different buffer zones. (a) Patch Density; (b) Edge Density; (c) Landscape Shape Index; (d) Largest Patch Index; (e) Area-weighted Mean Euclidean Nearest-Neighbor Distance; (f) Area-weighted Mean Patch Fractal Dimension.

Patch density and edge density increased as the buffer distance increased (Fig 9A and 9B). The LPI was largest between 10 to 30 km buffer zones, indicating a more complicated landscape shape in these zones as compared to others (Fig 9C). On the other hand, the largest patch index was smaller between 15 to 30 km buffer zones compared to other buffer zones (Fig 9D). As such, the largest patch in the 15 to 30 km buffer zones only occupies 4% to 10% of the landscape, whereas in the 5 km buffer zone it makes up 81% of the landscape (Fig 9D). Thus, the LPI indicates that urban landscape is fragmented into smaller patches in the 15 to 30 km buffer zones as compared to the others. The values of ENN_AM appear to be higher from the 15 to the 35 km buffer zones (Fig 9E). As a result, patches in between 15 to 35 km buffer zones are further apart or more isolated from each other, especially at 20 and 35 km buffer zones (Fig 9E). The FRACT_AM values are higher in between the 5 to 35 km, indicating that the patch level shape complexity is higher where it is closer to the urban center and reduced as it goes further from the urban center indicated by the increased buffer distance (Fig 9F).

**Fig 10:**
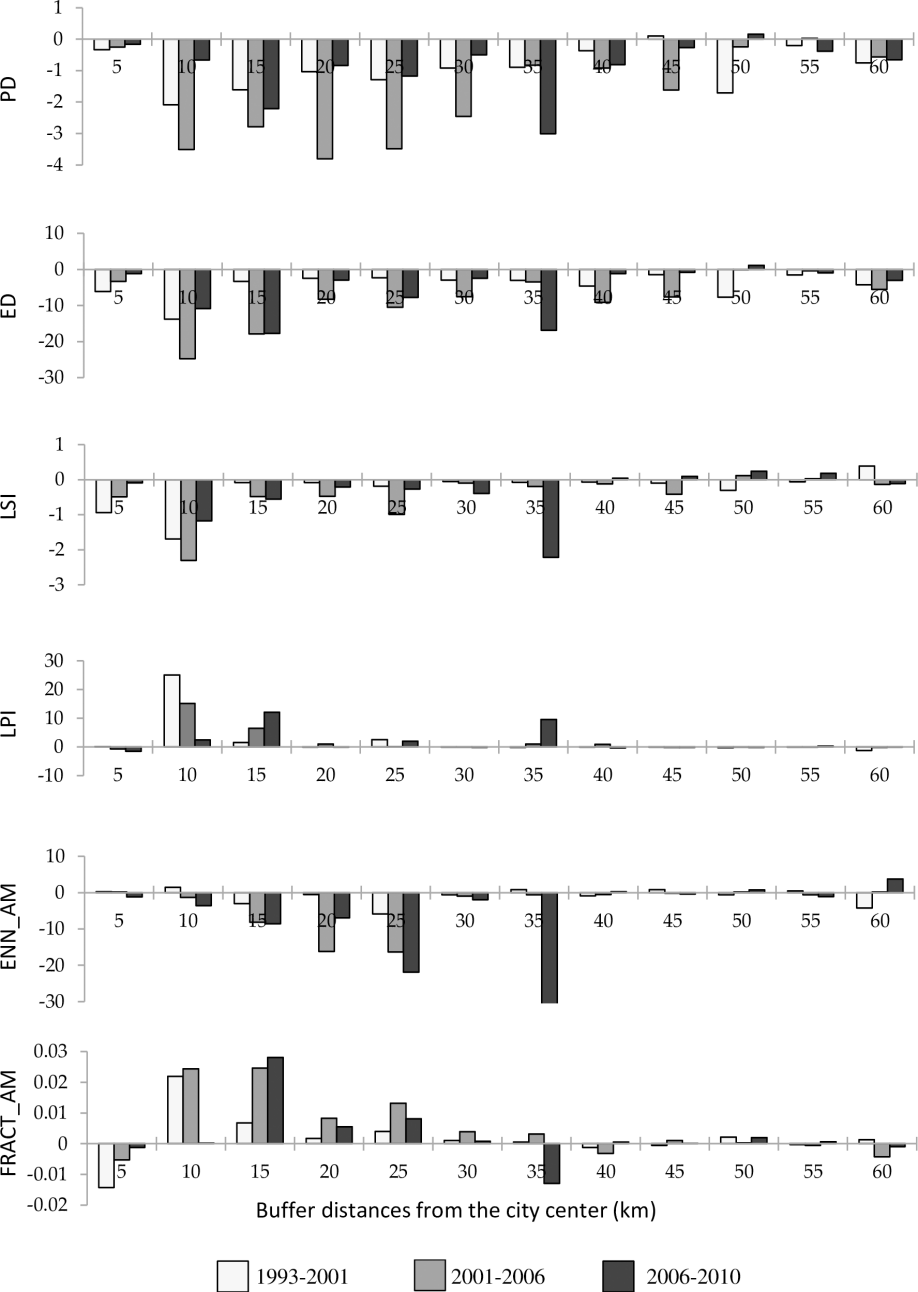
Changes in mean values of the patch metrics at different buffer zones over three time periods. The changes in each patch metric across the three time periods was computed as the Difference in spatial metric i = Spatial metric i (t_2_)–Spatial metric i (t_1_). For a given patch metric, an increase in its value from time one (t_1_) to time two (t_2_) leads to a positive difference and a decrease in its value from t_1_ to t_2_ results in a negative difference.

For each patch metric, trend and magnitude of the changes differed among the three time periods and across different buffer zones (Fig 10). Over the three time periods, the PD and ED decreased. These results suggest that the degree of landscape fragmentation has been decreasing over time. The LSI also decreased within the 5 to 35 km buffer zones. The decrease of the LSI in these zones indicates that over time the shape of the landscape becomes closer to the regular-square and circle shape. The LPI increased in the 10, 15, 25, and 35 km buffer zones while it remains quite stable in other locations. The increase of the LPI over time in these buffer zones reveals the prevalence of infilling and edge expansion during the urbanization process.

The ENN_AM has decreased over time in the 10 to 35 km buffer zones and it slightly fluctuates in other buffer zones. The decrease of ENN_AM in these buffer zones signified a decrease of patch isolation in these areas. The FRACT_AM has decreased at the 5 km buffer zone, indicating that over time the shape of the urban patches is becoming more regular with a simpler shape. Between the 10 to 25 km buffer zones, the patch shape has become more irregular and complex as indicated by the increase of FRACT_AM. The patch shape index at the 35 km buffer zone had slightly increased in the first two periods but then decreased in the third period, indicating a more regular shape of urban patches. Changes of FRACT_AM in other buffer zones are negligible as the result of low urban growth rates (Fig 4). Taken together the results indicated that major change in spatial metrics occurred from the 10 to 25 and at 35 km buffer zones which correspond to the faster pace of urbanization in these areas.

## Discussion

### Varying urban growth rates across buffer zones

Urbanized in Hanoi progressively increased over the 17 years from 1993 to 2010. The annual growth rate differed among the 12 buffer zones and three time periods. Specifically, the annual urban growth rate was highest at the 10 to 25 and 35 km buffer zones and in the periods 2001–2006 and 2006–2010. Based upon these spatiotemporal patterns we were able to identify a hot zone of urbanization that follows the wave-like growth pattern consistent with wave theory that predicts a hot zone of growth moving outwards from the city core with a particular periodicity [46]. Though the wave pattern was unclear in the first period (1993–2001), due to low urbanization, the relationship between the growth area and the distance factor clearly displayed a wave pattern in the second (2001–2006) and third period (2006–2010) (Fig 5). Spatiotemporally, the hot zone, as indicated by the wave peak, peaked at 10 km buffer zone between 2001 and 2006 and shifted to 15 km buffer zone between 2006 and 2010. As the appearance of new growth centers occurred, some hotspots would occur in further area, creating multi-peaked patterns, such as the peak at 25 km buffer zone where Hoa Lac High-tech zone and two industrial park projects—Quoc Oai and Quang Minh—were laid down their foundation between 2001 and 2006, and the peak at 35 km buffer zone where Son Tay—a new town of Hanoi is being modernized as a satellite city and a recreation center. Identifying these hotspots can provides important information for urban planning and development.

### Diffusion and coalescence phases of urban growth

The idea of alternating urban growth phases has a long history of support [5, 13, 15, 47, 48]. Contemporary research suggests that the urban growth process may exhibit two alternate diffusion and coalescence phases, and that spatial metrics can be used to quantify this sequential process [15, 18, 49]. The urbanization process of Hanoi during the 17 years we evaluated has also experienced this oscillation. However, we noticed that the switches between diffusion and coalescence phases were not concurrent among buffer zones as some may have shorter or longer oscillation cycles, depending on the urban growth rate in each buffer zone. For example, the urban growth rate in the 10 km buffer zone was higher than that in the 5 km buffer zone (Fig 5). As the result, the 10 km buffer zone quickly shifted from the diffusion to coalescence phase between the second and third time periods while the 5 km buffer zone still continued with the coalescence phase throughout the whole study period (Fig 8). Thus, over 17 years, the coalescence-diffusion cycle had been repeated in the 10 km buffer zone, meanwhile the 5 km buffer zone had not completed one cycle yet. It is important to note that the two-phase diffusion-coalescence concept can be easily misleadingly as overly simplistic because, in reality, all three urban growth modes occur simultaneously in the same landscape [19].

### Changes in spatial patterns along the rural-urban gradient

The spatial metrics effectively described the structure and changes of the urbanization occurring in Hanoi landscape. The urban core had lower values of PD, ED, LSI, and ENN_AM, but higher values of LPI and FRACT_AM (Fig 9). In addition, high urbanization zones were observed between 10 and 35 km buffer zone (Fig 10), consistent with the urban growth rate (Fig 5). The urbanization zones of high growth rate experienced a decrease of certain spatial metrics such as PD, ED, LSI, ENN_AM and an increase of LPI, supporting diffusion-coalescence theory [11, 15]. However, we did find an increase of FRACT_AM in the high urbanization zone which differs from diffusion-coalescence theory. The increase of FRACT_AM may indicate the absence or weak land use planning activities of the city resulting in uncoordinated development. The urban growth characteristic of Hanoi differ from findings in Yangtze River Delta (YRD) in China from 1979 to 2008 [19] and in a study of 120 cities worldwide from 1990 to 2000 [50], which found that the high urbanization rates tend to increase the values of PD, ED, and LSI. These differences suggest that spontaneous growth in YRD in China, driven by several cities at county and prefectural level, shared a significant amount of growth over time whereas spontaneous growth is the least growth mode found in Hanoi over 17 years period. The low spontaneous development in Hanoi often indicated a poor social infrastructure, a bad connection between the periphery and the city center and the lack of public services [51]. Most of the urban growth in Hanoi was within or adjacent to existing residential areas. Except in the 5 km buffer zone, where the urban landscape is essentially saturated with buildings, the high urbanization zones in Hanoi can be considered as in the inception phase of urbanization. In the next several years, when the existing residential areas fill up and old infrastructures becomes overloaded, the spontaneous growth/leapfrogging is likely to become the dominant growth form and changes in the landscape are likely to be similar to what has occurred in YRD in China [19] or general landscape changes of 120 cities worldwide [50]. Our findings supported the suggestion that urbanization tends to decrease the spatial heterogeneity of landscapes, resulting in homogenization of urban landscape structure [50]. However, while this suggestion is true in the long run or as a final state of urbanization, during the urbanization process, the urban landscape structure change will follow a wave-like pattern where the coalescence and diffusion are simultaneously happening at different locations or switching each other in the same location.

### Planning implication

The urban growth dynamics and forms of urbanization we identified provide essential information for land use management and planning authorities. For instance, there has been a lengthy discussion on whether it is more beneficial to manage for compact or diffuse cities [44] as there are both advantages and disadvantages in each approach. Because Vietnam’s urban areas are currently being developed it is critical to consider what form it should take. More diffuse urbanization may lead to leaving native habitats that are networked and thus can support diverse species and that provide accessible green spaces throughout the city [44]. Compact cities, in contrast, are better in terms of land use efficiency and energy consumptions [52], but may have reduced ecosystem functions and services [53]. The compact model has been widely adopted as a planning approach in developed countries, especially in European countries [54]. However, compact cities often require a strong governmental leadership for policy implementation and involve a radical change in the lifestyle of residents, such as enhancing the saving of resources, minimizing waste discharge, and recycling. These pose a challenge for developing countries to adopt a compact growth model and to achieve in a sustainable manner. Past studies have suggested that cities around the world are becoming less dense as they grow [55]. But a recent World Bank study on urban expansion in East Asia found that population is growing faster than urban footprints in most East Asian cities [56] and the findings from Hanoi supports this conclusion. The compact growth (high density) of cities in many developing countries does not draw them to sustainable growth. Instead, these are the consequences of large families living together due to high housing price, housing shortage, poor infrastructure, and public transportation that restrict population migration to expand to surrounding regions [57]. Therefore, opting for the compact or diffuse model of urban growth needs to be based on past and current data of urbanization as well as the role government will play in the decisions and management.

Within Hanoi specifically, the different urban growth phases also provide important information for policymakers and planning authorities. On the one hand, where urbanization is in the coalescence phase, the urban form will be more compacted by the dominance of infilling growth process. As such, planning authorities should be aware of the surrounding areas that will likely receive great pressure from land transformation. On the other hand, where urbanization is in the diffusion phase, urban growth tends to disperse far from the existing urban areas causing landscape fragmentation, biodiversity losses, and disruption of ecosystem functions. Green belt and remnant forest areas are critical for providing habitats and maintaining ecosystem functions [58, 59], indicating that maintaining natural habitats, green belts, and remnant forest areas would help to mitigate the negative impacts of the urbanization and should be highly prioritized in any urban development plans.

## Conclusions

Cities in Vietnam have rapidly urbanized since the country adopted the economic reform in 1986. In the case study of Hanoi, the built-up land has exponentially expanded, especially between the 10 to 35 km buffer area from the city center. The urban growth dynamics were described by relative dominance of infilling, edge expansion, and spontaneous growth modes across the landscape in which infilling and edge expansion were the dominant types. Our observation of the Hanoi urbanization process supports diffusion-coalescence phase dynamics that show an oscillation cycle resulting in the regular shift of growth hot zones that can provide important information for urban modeling and prediction.

While our study revealed a clear link between empirical measures and the theoretical model of urban growth, we did not evaluate the factors that led to such growth. Urban growth is often driven by combination of factors, such as topography, planning policy, and the initial conditions of the city. Thus, while such factors may well be responsible for the observed changes in Hanoi, they were beyond the scope of the current and provide a fruitful avenue for future research.

The approach we have taken has great potential for other cities in Vietnam and around the world. In particular, our approach can be useful to describe the spatiotemporal patterns of urban growth which can then be used to evaluate the land use planning policy as well as to guide future land use planning activities. Future research in Hanoi should investigate in understanding the underlying causes and ecological impact of landscape transformation due to urbanization processes.
